# Alterations in the Expression Profile of Serum miR-155, miR-223, miR-17, miR-200a, miR-205, as well as Levels of Interleukin 6, and Prostaglandins during Endometritis in Arabian Mares

**DOI:** 10.3390/vetsci8060098

**Published:** 2021-06-04

**Authors:** Sally Ibrahim, Mohamed Hedia, Mohamed O. Taqi, Mohamed K. Derbala, Karima Gh. M. Mahmoud, Youssef Ahmed, Sayed Ismail, Mohamed El-Belely

**Affiliations:** 1 Department of Animal Reproduction and AI, Veterinary Research Division, National Research Centre, Dokki, Giza 12622, Egypt; karimamahmoud@yahoo.com (K.G.M.M.); yfahmed54@yahoo.com (Y.A.); 2 Department of Theriogenology, Faculty of Veterinary Medicine, Cairo University, Giza 12211, Egypt; mohammedhedia@cu.edu.eg (M.H.); sayed.ismail@vet.cu.edu.eg (S.I.); elbelely@cu.edu.eg (M.E.-B.); 3 Central Laboratory for Agricultural Climate, Agricultural Research Centre, Ministry of Agriculture and Land Reclamation, Dokki, Giza 12311, Egypt; mohamed.o.taqi@gmail.com; 4 Diagnostic Imaging and Endoscopy Unit, Animal Reproduction Research Institute, Giza 12556, Egypt; mohamed_equine@yahoo.com

**Keywords:** serum miRNA, mares, endometritis, interleukin 6, prostaglandins, age

## Abstract

So far the intimate link between serum microRNA (miRNA) and uterine inflammation in mares is unknown. We aimed (I) to investigate expression profile of eca-miR-155, eca-miR-223, eca-miR-17, eca-miR-200a, and eca-miR-205 (II) and to measure concentrations of interleukin 6 (IL-6), and prostaglandins (PGF_2α_ and PGE_2_) in serum of mares with healthy and abnormal uterine status (endometritis). This study was conducted on 80 Arabian mares: young (4–7 years), and old (8–14 years). Mares were divided into 48 sub-fertile (endometritis) and 32 fertile (control) at stud farms. Serum was collected for measuring IL-6, PGF_2α_, and PGE_2_, as well as miRNA isolation and qRT-PCR. Concentrations of IL-6, PGE_2_, and PGF_2α_ were higher in mares with endometritis compared to control. Age of mares had a remarkable effect on IL-6, PGE_2_, and PGF_2α_ concentrations. Relative abundance of eca-miR-155, eca-miR-223, eca-miR-17, eca-miR-200a, and eca-miR-205 was higher in both young and old mares with endometritis. We noticed that eca-miR-155, eca-miR-223, eca-miR-200a, and eca-miR-205 revealed higher expression level in old than young mares with endometritis. This is the first study that has revealed the changes in cell free miRNA and serum inflammatory mediators during endometritis, and these findings could be used for a better understanding the pathophysiology mechanisms of endometritis in equine.

## 1. Introduction

For years, equine endometritis has been considered as the most important cause of infertility in horses [1]. Infectious endometritis is the first cause of equine subfertility and the third most common disease affecting horses [2]. Endometrial infections are directly responsible for decreasing conception rates and indirectly disrupt reproductive outcomes leading to early embryonic loss, abortion and delivery of intrauterine infected foals [3].

An inflammatory response against uterine pathogens is important to control and eliminate the uterine harmful infections, which seems to be the main cause of subfertility and failure of conception [1]. The initial defense mechanisms against endometrial pathogens are directly evoked as an inflammation, which subsequently activate innate and humoral immune responses in the mares [4] via series of cytokines and chemokines. Cytokines have an important role in a wide range of reproductive related processes. There are several types of cytokines, where each cytokine is responsible for multiple cellular tropism in an array of different organs and their response showed a different manner according to cell type [5]. There have been several studies investigating the temporal pattern of endometrial cytokine expression in mares susceptible and resistant to persistent endometritis. Fumuso et al. [6] reported that endometrial IL-6 mRNA increased 24 h after insemination in susceptible compared with resistant mares. In addition, Christoffersen et al. [7] investigated that 3 h after intrauterine infusion with *E. Coli* elicited marked increased endometrial IL-6 mRNA expression in resistant compared with susceptible mares. Woodward et al. [8] demonstrated that the expression of endometrial IL-6 mRNA was higher after 6 h in resistant mares. Previous studies reported that the expression of pro-inflammatory cytokines IL-6 and prostaglandins (PGF2α and PGE2) in the uterine tissue samples were associated with the development of endometritis in cows [9] and mares [6,8]. Prostaglandins play an important role in the equine endometrium during different stages of the estrous cycle [10], as well as in inflammatory processes [11]. Furthermore, the dysregulation in their secretion may perturb uterine functions [12].

The proper clearance of excess sperm cells, microorganisms, seminal plasma, and other debris from lumen of uterine tissues is an essential step for qualifying uterine milieu for implantation after embryo arrival [13]. This step is a complex and influenced by the host’s immune system as well as epigenetic regulation [14]. Nothing is known about the expression pattern of serum miRNA and post-transcriptional regulation of inflammatory immune response genes in mares with endometritis. Recently in equine species, many studies revealed that serum miRNA could be used as non-invasive biomarkers either for normal physiological condition (early pregnancy) or disease condition (sarcoid disease) [15,16]. The miRNA are small non-coding RNA molecules that act as post-transcriptional regulators of gene expression by inhibiting translation or degrading mRNA through partial or complete base pairing with three prime untranslated region (3′-UTR) of the target mRNAs [17]. They are expressed in different cells and tissues, in order to regulate different mechanisms of developmental as well as physiological processes [16,18]. Furthermore, an extended inflammatory response due to aberrant miRNA expression is likely to impair fundamental cellular processes of the endometrium, affecting uterine receptivity, folliculogenesis, oocyte maturation, and ovulation, finally leading to reduced fertility [12,19]. It was demonstrated that miRNA plays a vital role in regulation of the inflammatory immune response genes in mammalian female reproductive health [20]. For instance, miR-155, miR-223, miR-215, miR-17 were differentially expressed in response to induced inflammation by *Lipopolysaccharides* (*LPS*) [12,15,21]. Furthermore, miR155 is indicated to be endotoxin-responsive genes [22]. It was also reported that miR-200, miR-205, miR-215, and miR-17 were highly discriminated between cows with metritis and normal ones [23]. Moreover, eca-miR-155, eca-miR-223, eca-miR-17, eca-miR-200a, and eca-miR-205 were shown to be targeting the inflammatory immune response genes such as TNF receptor associated factor 6 (TRAF6), tumor necrosis factor alpha (TNFα), interleukin 6 (IL6), interleukin 8 (IL8), and interleukin 10 (IL10) [12]. 

So far, the expression pattern of free serum miRNA is unknown during endometritis in equine species. We therefore hypothesize that identification and quantification of some candidate serum miRNA from mares with endometritis might serve as useful step for a better understanding the molecular regulation (post-transcription level) of endometritis in mares. Moreover, any alteration in the endometrial health status might be accompanied with series of pathophysiological changes, which subsequently dysregulate expression pattern of serum miRNA as well as increase the serum levels of inflammatory biomarkers as IL-6, PGF_2α_, and PGE_2_. Thus, the current study aimed (I) to investigate the expression profile of eca-miR-155, eca-miR-223, eca-miR-17, eca-miR-200a, and eca-miR-205, and (II) to measure the concentrations of IL-6, PGF_2α_, and PGE_2_, in serum of young and old aged mares with healthy and abnormal uterine status (endometritis).

## 2. Materials and Methods

### 2.1. Chemicals

All chemicals and reagents were obtained from Qiagen (Hilden, Germany), Thermo Fisher Scientific (Wilmington, NC, USA), unless otherwise stated.

### 2.2. Ethical Approval for Use of Animals

The present study was approved by the Ethical Use and Animal Care Committee of Faculty of Veterinary Medicine, Cairo University (VET. CU 16072020166).

### 2.3. Animals and Management

This study was conducted on 80 Arabian mares: young (4–7 years), as described by Carnevale and Ginther [24] and Hurtgen [25], and old (8–14 years). These mares were divided into 48 sub-fertile mares (young (n = 16), old (n = 32)) suspected of endometritis (diseased group) and 32 fertile mares (control: young (n = 24), old (n = 8)) not suspected of endometritis that served as control group between November 2019 and April 2020 at a number of stud farms nearby Giza, Egypt. Uterine swabs and blood samples were collected only once from each mare 1–3 days before insemination during the estrus, after the owner’s permission. The selected mares experienced normal physical and vital signs where the normal range of vital signs including rectal body temperatures (37.5–38.5 °C), heart rate (36–40 beats/min), respiratory rate (8–15 breaths/min), capillary refill times (1–2 s), and a moist with a healthy pink color mucus membrane of the buccal cavity. An orthopedic examination was also performed to exclude mares with lameness or active laminitis. None of the mares had dystocia, retained fetal membranes or problems during puerperium. Additionally, none of the mares was in foal heat. All animals were submitted to transrectal ultrasonographic (US) uterine examination using real-time B-mode machine (Esaote Mylab30-Maastricht, The Netherlands) equipped with 5–7.5 MHz linear-array transducer. 

Here in the current work, the criteria for mares to be enrolled in the diseased group (endometritis) were that they had been bred three or more times unsuccessfully in the breeding season, or had a history more than one year of reproductive failure. In addition, two or more of the following criteria on a checklist were present: US scanning showed abnormal fluid in the uterus (echogenic or ≥2 cm in diameter), positive endometrial cytology, and pathogenic bacterial and/or fungal growth, as shown before [26]. In addition, healthy mares exhibited normal breeding history, normal uterine US appearance, and did not show any pathogenic microbial growth for the uterine samples, as well as negative cytology data.

### 2.4. Blood and Endometrial Swabs Sampling

Blood samples were collected from the jugular vein into untreated vacutainer tubes and the tubes were maintained for 20 min at room temperature in an inclined position. The samples were subsequently centrifuged at 3000× *g* for 10 min (4 °C) until clear serum was separated. Afterwards, serum was divided into two portions: the 1st part was kept at –20 °C for measuring IL-6, PGF_2α_ and PGE_2_ concentrations, and the 2nd part was kept at –80 °C for RNA isolation. In order to prevent the degradation of prostaglandins; serum was added into a 1% stabilizing solution 0.3 M ethylenediaminetetraacetic acid (EDTA) (Sigma) and 1% aspirin (Sigma).

Endometrial swabs were collected as described before [27] using a sterilized double guarded uterine swab (Minitub GmbH, Bavaria, Germany). Briefly, after removing feces from the rectum, the tail was bandaged, and then vulva and perineum were cleaned with iodopovidine (Betadine, EGIS, Warsaw, Poland), rinsed three times with water, and dried with a paper towel. The tip of the swab was held and covered in the palm, and using a slight rotatory movement, the hand passed into the vagina towards the external cervical os, and then the index finger was passed gently into the external cervical os followed by the uterine swab. After passing the cervical canal, the cotton swab pushed forward through the outer then the inner guards to be contacted with the endometrium then gently rotated for 10–15 s. Finally, the swab was retracted back inside the inner guard then into the outer guard and the swab directly immersed into Cary-Blair transport medium (Oxoid, Lenexa, KS, USA).

### 2.5. Cytological Examination

After collection of uterine smears as described above, the cytological samples were fixed and stained with a special commercial cytological stain, Papanicolaou method (Biodiagnostic, Egypt) according to the instruction’s recommendation within two hours at the laboratory. Samples were evaluated in regard to quality of cell morphology, cellularity, number of inflammatory cells per 400× field, as well as any other remarkable features (Zeiss Axioskopmicroscobe, Carl Zeiss, Thornwood, NY, USA) [28]. Uterine samples were considered positive for endometritis if the amount of PMNs was greater than 2%, as described before [29].

### 2.6. Microbial Culturing 

Immediately after immersing the uterine smears into the transportation media, samples were transported to the laboratory for further microbial analysis. Both bacterial and fungal culturing protocols were carried out to identify the pathologically infected mares showing endometritis according to the general guidelines [30].

### 2.7. Serum IL-6, PGF_2α_, and PGE_2_ Estimation

Serum concentrations of IL-6 were determined by horse IL-6 ELISA kit (SunLong Biotech Co., LTD, Zhejiang, China) and used according to the manufacturer’s instructions. The assay sensitivity and range were 0.5 pg/mL and 1.6 pg/mL to 100 pg/mL, respectively. For PGF_2α_ measurement, the commercial PGF_2α_ high sensitivity horse prostaglandin F_2_ alpha ELISA kit (SunLong Biotech Co., LTD, Zhejiang, China) was used and run according to the manufacturer’s instructions. The assay sensitivity and range was 0.5 pg/mL and 3 pg/mL to 210 pg/mL. For PGE_2_ measurements, the commercial PGE_2_ high sensitivity horse prostaglandin E_2_ ELISA kit (SunLong Biotech Co., LTD, Zhejiang, China) was used and run in accordance to the manufacturer’s instructions. The assay sensitivity and range were 0.1 pg/mL and 0.8 pg/mL to 50 pg/mL, respectively. The inter- and intra-assay coefficients of variation were 8.6% and 5.5% for IL-6, 6.6% and 10.79% for PGF_2α_, and 6.9% and 12.2% for PGE_2_.

### 2.8. In Silico Analysis for the Selected Candidate miRNA

The miRNA prediction tools such as: DIANA-microT v3.0 (http://diana.cslab.ece.ntua.gr/microT/, accessed on 4 April 2021) (Volos, Greece) and miRecords (LC Sciences, Houston, TX, USA) (http://mirecords.biolead.org/, accessed on 4 April 2021) were used for selection miRNAs according to their potential relevance for targeting inflammatory immune response in mammalian uterine tissue at least in four different search algorithms. We found that eca-miR-155, eca-miR-223, eca-miR-17, eca-miR-200a, and eca-miR-205could be selected as potential targets. Moreover, these targets exhibited different expression patterns in response to inflammation [12,24,31,32].

### 2.9. Serum miRNA Isolation, cDNA Synthesis, and Quantitative Real-Time PCR (qRT-PCR)

For purification of cell-free miRNAs, we used a miRNeasy serum/plasma kit (Qiagen, Hilden, Germany). An amount of 200 μL of thawed serum samples on ice was used for purification of total RNA according to manufactures protocol. During purification steps, 3.5 μL of lyophilized *C. elegans* miR-39 miRNA mimic (miRNeasy Serum/PlasmaSpike-In Control), (Qiagen, Hilden, Germany) were added, at concentration 1.6 × 10^8^ copies/μL. In order to elute cell-free RNA, 14 μL of RNase-free water was added to the center of the spin column membrane. Afterwards, the purified serum free total RNA was kept at −80 °C. The concentration of total RNA was checked by Nano-drop 2000/c (Thermo Fisher Scientific, Waltham, MA, USA). We selected samples with absorbance ratios above 1.7. Furthermore, the integrity of RNA was evaluated by denaturing 1.5% agarose gel electrophoresis and ethidium bromide staining. Around 10 ng of total RNA was reverse transcribed using MultiScribe reverse transcriptase (Thermo Fisher Scientific, Wilmington, NC, USA), and RT primers (Thermo Fisher Scientific, Wilmington, NC, USA) were performed separately for each miRNA according to the supplier’s instructions. Real-time PCR was done in a final volume of 10 μL, using 0.7 μL of RT product, 0.5 μL of specific primers with probes (Table 1), and TaqMan Universal PCR Master Mix II (Thermo Fisher Scientific, Wilmington, NC, USA). Amplification was performed with initial denaturation for 10 min at 95 °C, followed by 40 cycles of 15 s at 95 °C and 60 s at 60 °C with Stratagene Mx3000P (Agilent Technologies, Waltham, MA, USA), PCR reactions were performed in quadruplicates. The data were analyzed by the comparative threshold cycle (ΔCt) method and normalization was performed using geometric means of cel-miR-39-3p, eca-miR-195, and U6.

### 2.10. Statistical Analysis

The NormFinder was used to select the most stable reference gene for normalization miRNA data [33]. The raw data of fluorescence values (Rn) were imported into PCR Miner in order to calculate efficiency [34]. The normal distribution was checked via Shapiro-Wilk test and Gaussian distribution, and all data passed normality tests (Alpha ≤ 0.05), (GraphPad Software, Inc., San Diego, CA, USA). The expression of selected miRNAs and the levels of serum IL-6, PGF_2α_, and PGE_2_ were analyzed using two-way ANOVA, followed by Sidak’s multiple comparisons test. The values shown in graphs are presented as the mean±standard error of the mean (S.E.M.) of at least five independent experiments each done in quadruplicate (i.e., it was at least five biological replicates per each group (control and diseased), and these biological replicates were sub-divided into technical replicates). *p* values ≤ 0.05 were considered statistically significant. GraphPad Prism 9.0 (San Diego, CA, USA) was used to perform statistical analysis as well as to generate bar graphs.

## 3. Results

### 3.1. Cytological and Microbial Findings

Control mares were negative for the presence of uterine neutrophils (Supplementary Figure S1a–c). Forty-eight sub-fertile mares were positive for microbiological culture. A total of 95.83% of isolates were bacterial while the rest (4.17%) were fungal isolates. The prevalence of microbial isolates were: Beta-hemolytic streptococci (33.33%), Staphylococcus aureus (20.83%), Staphylococcus epidermidis (14.85%), Escherichia coli (16.67%), Klebsiella spp. (10.42%), and Candida albicans (4.17%) (see Supplementary Figure S2). 

### 3.2. Dynamic Pattern of Serum IL-6, PGE_2_, and PGF_2α_ in Mares with Endometritis (Diseased) Compared to Control Ones

**Figure 1:**
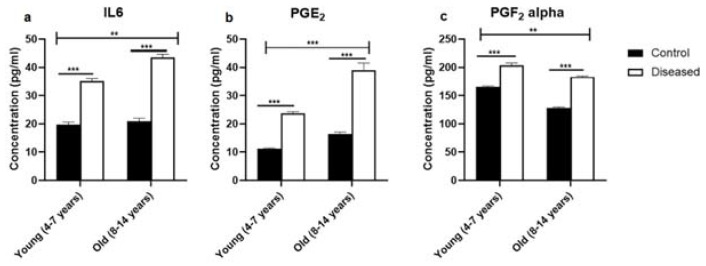
The levels of IL-6 and prostaglandins (mean ± SEM) in serum of mares (young (4–7 years) and old (8–14 years)) with endometritis compared to control ones. (**a**) The serum concentration of IL-6. (**b**) The level of PGE_2_ in serum. (**c**) The serum level of PGF_2α_. Statistical significance was defined as values of *p* < 0.05. Statistical differences among groups are marked with asterisks (** *p* < 0.01, *** *p* < 0.001).

**Figure 2:**
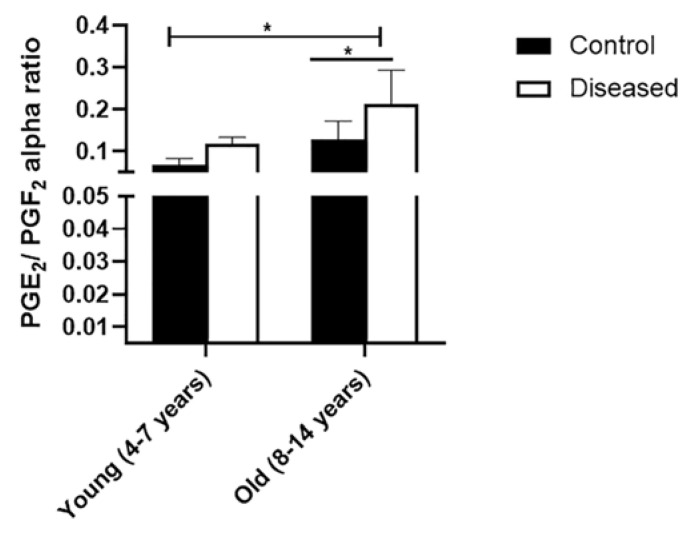
Ratio (mean±SEM) of serum PGE_2_/PGF_2α_ concentrations in serum of mares (young (4–7 years) and old (8–14 years)) with endometritis compared to control healthy ones. Statistical significance was defined as values of *p* < 0.05. Statistical differences among groups are marked with asterisks (* *p* < 0.05).

Serum concentrations of IL-6 were higher (*p* < 0.001) in diseased mares (both young and old) compared to the control ones. In the diseased mares, there was a remarkable increase (*p* < 0.01) in the serum IL-6 levels in the old compared to the young ones (Figure 1a). In the same sense, serum concentrations of PGE_2_ displayed higher values (*p* < 0.001) in the diseased mares (both young and old) compared to the healthy ones. Old healthy and diseased mares showed increased values (*p* < 0.001) of serum PGE_2_ compared to the young ones (Figure 1b). Serum levels of PGF_2α_ recorded a marked increase (*p* < 0.001) in the diseased mares (both young and old) compared to the healthy ones. In addition, young mares (control and diseased) exhibited a higher value (*p* < 0.01) of the serum concentration of PGF_2α_ compared to the old mares (Figure 1c). In addition, PGE_2_ to PGF_2α_ ratio was significantly (*p* < 0.05) higher in the young and old mares with endometritis compared to the control mares (Figure 2).

### 3.3. Fold Regulation of Serum miRNA in Mares with Endometritis Compared to Normal Healthy Ones

**Figure 3:**
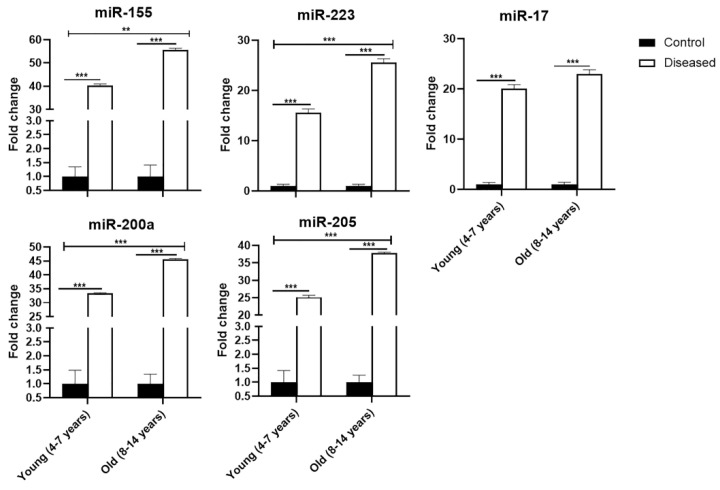
Expression profile of eca-miR-155, eca-miR-223, eca-miR-17, eca-miR-200a, and eca-miR-205in serum of mares (young (4–7 years) and old (8–14 years)) with endometritis compared to control ones. Bars are presented as mean ± SEM. Asterisk(s) represent statistical significance; ** *p <* 0.01, *** *p <* 0.001.

In Figure 3, the relative abundance of eca-miR-155, eca-miR-223, eca-miR-17, eca-miR-200a, and eca-miR-205 was higher *p* ≤ 0.001) in both young and old diseased mares, compared to control healthy mares (young and old). In old mares, eca-miR-155, eca-miR-223, eca-miR-200a, and eca-miR-205 revealed higher (0.001 ≤ *p* ≤ 0.01) expression than young diseased mares, as well as in all control mares.

Taken together, there were significant (0.001 ≤ *p* ≤ 0.05) interactions among groups: control healthy young, control healthy old, diseased young, and diseased old mares, as shown by Sidak’s multiple comparisons test.

## 4. Discussion

As far as we know, this is the first report that measures the serum concentrations of IL-6, PGF_2α_, and PGE_2_ in mares with endometritis compared to the healthy Arabian mares. In mares with endometritis the serum concentrations of IL-6, PGF_2α_, and PGE_2_ were significantly higher compared with mares that did not suffer from endometritis. In addition to these clear points, the present study recorded that the inflammatory response could be different, with respect to the systemic cytokines, according to the age of the mares. It is well documented that increased age is associated with increased susceptibility to endometritis [35].

In the present study, the serum concentrations of IL-6 were markedly increased in mares with endometritis compared with healthy mares. Serum concentrations of IL-6 showed a significant increase in subclinical and clinical endometritis in cows [36] and ewes [37]. It is well-known that IL-6 is considered as the most important pro-inflammatory cytokine through the inflammation cascade. In addition, IL-6 has a supporting and modulatory role during the inflammation of the tissues, where IL-6 stimulates and potentiates the immune response as well as the action of the other cytokines as IL-10 [38,39]. The IL-6 is a soluble mediator with a pleiotropic effect on inflammation, immune response, and hematopoiesis, whereas it triggers synthesis of acute phase proteins (CRP, serum amyloid A, etc.) [40,41]. Moreover, IL-6 has a vital role on acquired immune response via stimulation of antibody production and T-cell development. Additionally, it promotes differentiation or proliferation of several non-immune cells [42]. 

Prostaglandins have a great crucial role during inflammation. PGs are considered as main modulators for the inflammatory response against pathogens, where they contribute to the control of the pathogens and alleviate its side effects in different species [43]. In the present study, serum concentrations of PGF_2α_ and PGE_2_ were markedly increased in mares with endometritis compared to healthy mares. The considerable increase in the concentration of these biomarkers is an indicator of strong response of inflammatory cells against inflammatory status of the endometrial cells in mares [7]. Furthermore, the uterine concentrations of PGE_2_ showed a remarkable increase 30 min after intrauterine bacterial inoculation in mares [3]. Interestingly, PGE_2_ is a potent uterine vasodilator, which are responsible for hyperemia of uterine blood vessels and chemotactic pattern for immune cells against uterine pathogens [44].

Here in the current study, we just measured the levels of IL-6 and PGs in serum, but we did not isolate total RNA from leukocytes and/or uterine biopsy, as was performed by Christoffersen et al. [7] or Woodward et al. [8]. Furthermore, we did not study different time-points of secreted cytokine and PGs, as well. Our results were in agreement with Nasreldin et al. [38], who revealed the association between endometritis (clinical and subclinical) and inflammatory cyrokines in serum. In previous research works that were done in mares, they only investigated the expression of these mediators in uterine biopsy or their level in cell culture supernatant [7,8,12]. 

No doubt that proper uterine function is regulated by different mediators such as miRNA [45], which is responsible for activation or inhibition certain target genes, depending on cell signaling. Uterine infection does not only affect female fertility by perturbing uterine function, but also could prolong ovarian cycle [46]. Therefore, to tackle the continuing fertility problems associated with uterine inflammation, understanding the molecular regulatory mechanisms associated with the inflammatory immune response is crucial to design appropriate therapeutic drugs [47]. Among the many molecular marks, the free serum miRNA expression patterns could be potential diagnostic as well as prognostic indicators of mares affected by endometrial inflammation. Accumulative studies revealed that miRNA could be used as diagnostic as well as prognostic biomarkers, through investigation their expression pattern between control and diseased ones [48,49,50]. Herein, there were a profound over-expression ineca-miR-155, eca-miR-223, eca-miR-17, eca-miR-200a, and eca-miR-205 in both young and old diseased mares, compared to control healthy mares (young and old). These findings are in agreement with previous studies in cattle, which revealed host cells responses to infection via activation the inflammatory immune response mediators, in order to overcome infection [12,20,24]. These inflammatory mediators could induce aberration of miRNA expression, which might be associated with an imbalance between pro-inflammatory and anti-inflammatory mediators [24,51,52]. Interestingly, there was a clear influence of mare age on the expression pattern of serum microRNA. On the other hand, old diseased mares showed higher expression level ofeca-miR-155, eca-miR-223, eca-miR-200a, and eca-miR-205 than young diseased mares, compared to control healthy ones. This might be due to increase fluid retention by mare age, and subsequently associated with clear changes in systemic immune response [36]. To the best of our knowledge, this is the first study to investigate the expression profile of eca-miR-155, eca-miR-223, eca-miR-17, eca-miR-200a, and eca-miR-205 in mares serum during endometritis.

## 5. Conclusions

To the best of our knowledge, this is the first study that revealed that equine endometritis was associated with clear changes in cell free miRNA and serum inflammatory mediators (IL-6, PGE_2_, and PGF_2α_). These findings could be a forward step for a better understanding the post-transcriptional regulation of endometritis in mares. Moreover, estimation of the serum concentrations of serum miRNA, IL-6, PGE_2_, and PGF_2α_ might be a promising recommended tool, during the breeding soundness examination in mares.

## Figures and Tables

**Table 1:** List of miRNA names, miRBase accession numbers, and mature sequences.

miR Name	Accession Number	Mature miRNA Sequence
eca-miR-223	MIMAT0013205	UGUCAGUUUGUCAAAUACCCCA
eca-miR-155	MIMAT0013182	UUAAUGCUAAUCGUGAUAGGGGU
eca-miR-200a	MIMAT0012909	UAACACUGUCUGGUAACGAUGU
eca-miR-17	MIMAT0013084	CAAAGUGCUUACAGUGCAGGUAG
eca-miR-205	MIMAT0012962	UCCUUCAUUCCACCGGAGUCUG

## Data Availability

The data that support the findings of this study are available from the corresponding author upon reasonable request.

## Supplementary Materials

The following are available online at https://www.mdpi.com/article/10.3390/vetsci8060098/s1, Figure S1: Uterine cytology in Arabian mares, Figure S2: Frequency of the microbial isolates in Arabian mares suffered from endometritis.
