# Supplementary material for: Alterations in the Expression Profile of Serum miR-155, miR-223, miR-17, miR-200a, miR-205, as well as Levels of Interleukin 6, and Prostaglandins during Endometritis in Arabian Mares
Source: Vet Sci. 2021 Jun 4;8(6):98. doi: 10.3390/vetsci8060098 (PMC8227551; doi:10.3390/vetsci8060098)
Supplement: Supplementary file 1 [file vetsci-08-00098-s001.zip › vetsci-1162797-supplementary.pdf]

Supplementary Material:

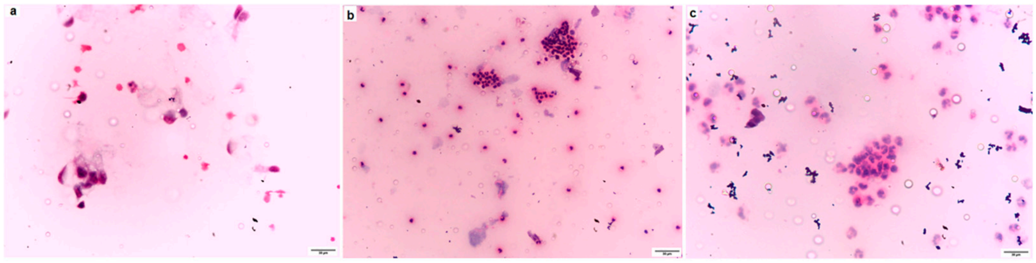

Supplementary Figure 1: Uterine cytology in Arabian mares. (A) Sample of normal healthy uterine cytology neither inflammatory cell nor bacteria were seen. (B) Cytological uterine sample from diseased mare showed diffused inflammatory cells and few colonies of bacteria. (C) Cytological uterine sample from diseased mare showed diffused inflammatory cells and many bacterial colonies (Pap. stain ×400).

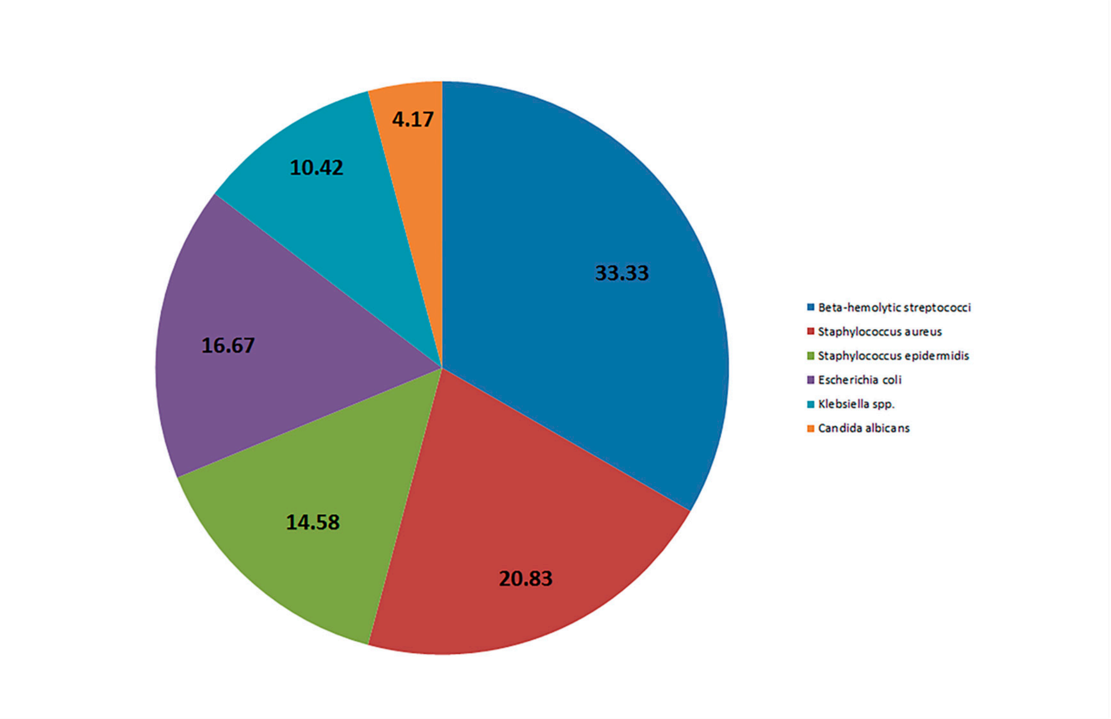

Supplementary Figure 2: Frequency of the microbial isolates in Arabian mares (n=48) suffered from endometritis; *Beta-hemolytic streptococci* (n=16), *Staphylococcus aureus* (n=10), *Staphylococcus epidermidis* (n=7), *Escherichia coli* (n=8), *Klebsiella spp.* (n=5), and *Candida albicans* (n=2).
